# Brief episodes of rapid irregular atrial activity (micro-AF) are a risk marker for atrial fibrillation: a prospective cohort study

**DOI:** 10.1186/s12872-020-01453-w

**Published:** 2020-04-10

**Authors:** Tove Fredriksson, Katrin Kemp Gudmundsdottir, Viveka Frykman, Leif Friberg, Faris Al-Khalili, Johan Engdahl, Emma Svennberg

**Affiliations:** Department of Clinical Sciences, Karolinska Institutet, Danderyd University Hospital, 182 88 Stockholm, Sweden

**Keywords:** Atrial fibrillation, Electrocardiogram, Micro-AF, Supraventricular ectopic beats, Screening, Supraventricular tachycardia

## Abstract

**Background:**

Short supraventricular tachycardias with atrial fibrillation (AF) characteristics are associated with an increased risk of developing AF over time. The aim of this study is to determine if presence of very short-lasting episodes of AF-like activity (micro-AF) can also be used as a marker of undiagnosed silent atrial fibrillation.

**Methods:**

In the STROKESTOP II study, a Swedish mass screening study for AF among 75- and 76-year-olds, participants with NT-proBNP ≥125 ng/L performed intermittent ECG recordings 30 s, four times daily for 2 weeks. Participants with micro-AF (sudden onset of irregular tachycardia with episodes of ≥5 consecutive supraventricular beats and total absence of p-waves, lasting less than 30 s) were invited to undergo extended AF screening using continuous event recording for 2 weeks. A control group of individuals without micro-AF was examined using the same ECG modalities.

**Results:**

Out of 3763 participants in STROKESTOP II who had elevated NT-proBNP levels and were free of AF, *n* = 221 (6%) had micro-AF. The majority of participants with micro-AF (*n* = 196) accepted further investigation with continuous ECG monitoring which showed presence of AF in 26 of them. In the control group (*n* = 250), continuous monitoring detected 7 new AF cases. Thus, AF was significantly more common in the micro AF group (13%) compared to the control group (3%), *p* < 0.001.

**Conclusions:**

Presence of short-lasting episodes of AF-like activity (micro-AF) indicates increased likelihood for undetected AF. Continuous screening therefore seems recommendable if a finding of AF would change clinical management.

**Trail registration:**

ClinicalTrials.gov, identifier: NCT02743416, registered April 19, 2016.

## Background

The European Society of Cardiology’s guidelines define atrial fibrillation (AF) as an irregular heart rhythm without P waves lasting a minimum of 30 s. The time criterion is based on consensus [1]. Individuals with AF are at increased risk of stroke, dementia, heart failure and death [2–6]. Oral anticoagulation (OAC) is associated with decreased risk of AF-associated morbidity and mortality [7]. Hence, early diagnosis of AF and adequate treatment is of importance to prevent AF complications. Less is known about the risk of shorter episodes of atrial fibrillation-like activity. It has been shown that supraventricular ectopic beats (SVEBs) and supraventricular tachycardias (SVTs) are associated with increased risk of AF and stroke over time [8–14]. Short irregular SVTs, morphologically similar to AF, with absence of P waves seem to be more likely to progress into AF than short regular SVTs with or without P waves [15]. In clinical practice short episodes of AF-like activity is frequently detected during telemetry and long-term ECG. Currently, there are no recommendations about how these patients should be managed or whether such episodes merit OAC treatment. The aim of our study is to determine if short episodes of AF-like activity, that we term micro-AF, are not only a risk factor for future AF, but are also markers for already existing undetected AF.

## Methods

This is a sub-study of STROKESTOP II, a Swedish mass-screening study for AF in 75- and 76-year-olds. The STROKESTOP II study protocol has been published previously [16]. In short, all inhabitants born in 1940 and 1941 in Stockholm County (*n* = 28,712) were randomised to a screening or a control group and invited for AF screening from April 2016 to March 2018.

All participants completed a health questionnaire including palpitation symptoms, gender, height, weight, use of OAC or antithrombotic treatment and earlier diagnoses (AF, diabetes, vascular disease, heart failure, hypertension and stroke/transient ischemic attack (TIA)). Blood pressure was measured in the supine position. All participants performed an index ECG using a 1-lead ambulatory handheld Zenicor II device (Zenicor Medical Systems, Stockholm, Sweden). Those without a prior diagnosis of AF and NT-proBNP ≥125 ng/L were asked to perform intermittent 30 s ECG recordings four times daily for 2 weeks and to make extra recordings if palpitations occurred, using the same handheld Zenicor device. For intermittent recordings, the Zenicor device has been validated with 92% sensitivity and 96% specificity for AF detection compared to a 12-lead ECG [17].

Participants without AF lasting at least 30 s (as per current definition) but with very short episodes of AF-like activity (micro-AF) during the intermittent ECG recordings in STROKESTOP II were invited to be part of this study.

**Fig. 1:**
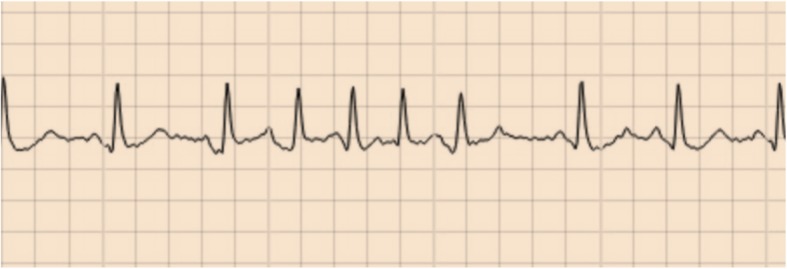
Micro-atrial fibrillation classification

We defined micro-AF as an irregular tachycardia of sudden onset with episodes of ≥5 consecutive supraventricular beats without P waves lasting less than 30 s, Fig. 1. Tachycardia was defined as an average heart rate of ≥100 beats per minute during the episode. P wave analysis and irregularity were determined by visual inspection, as it is commonly done in AF diagnostics. A computerised algorithm was used to identify ECGs with suspected micro-AF [18]. Trained nurses manually interpreted the episodes identified by the algorithm and marked suspected episodes of micro-AF. The findings were confirmed by the investigators (TF, JE).

All participants with micro-AF and an unmatched control group free from AF were invited to undergo continuous ECG monitoring in parallel or within a short period of time after their intermittent ECG monitoring. Participants in the control group were recruited consecutively during the last months of the STROKESTOP II study. The continuous monitor was a 1-lead event recorder, R-test 4 evolution (Novacor, Rueil Malmasion, France). The R-test 4 device has been validated compared to continuous ECG and has an automated algorithm with 92% sensitivity and a 87% specificity for AF detection [19]. As specificity is low, all AF episodes stored by the device were manually inspected. Participants were instructed to use the recording device for 2 weeks and had the possibility to press a symptom button if they experienced symptoms. All participants were asked to fill out a questionnaire with regards to AF-related symptoms during their 2 weeks ECG registration. R-test 4 evolution has a monitoring capacity of 32 days and can store a total of 60 min of ECG recording. If the storing capacity is exceeded, only the most characteristic AF episodes are kept. The device was programmed to store not only AF-suspicious activity but also other significant ECG findings, Supplementary Table 1.

A diagnosis of AF was confirmed if the duration of AF was ≥30 s. A cardiologist follow-up was offered to all participants with new AF or other significant arrhythmias.

Continuous variables that were non-normally distributed as well as ordinal data were reported as median with interquartile range (IQR) and analysed using Mann-Whitney U- test. Normally distributed continuous variables were reported as mean with standard deviations and analysed using independent samples T-test. Chi-square and Fisher’s exact test was used for proportions. Logistic regression was used for multivariable analyses of associations. Included in the analyses were significant variables in univariate analysis, excluding SVEBs, number of micro-AF episodes and micro-AF duration due to interaction with presence of micro-AF. The HATCH-score is used to predict progression of paroxysmal AF to more permanent forms and to predict new on-set AF [20]. Accessible variables included in HATCH-score (hypertension, stroke/TIA and heart failure) were considered potential confounders and were also included in the multivariable analysis. All tests were two-sided, and values of *p* < 0,05 were regarded as significant. All analyses were performed using IBM SPSS statistics, version 24 software (IBM SPSS Statistics, IBM Corp, Somers, NY).

## Results

**Fig. 2:**
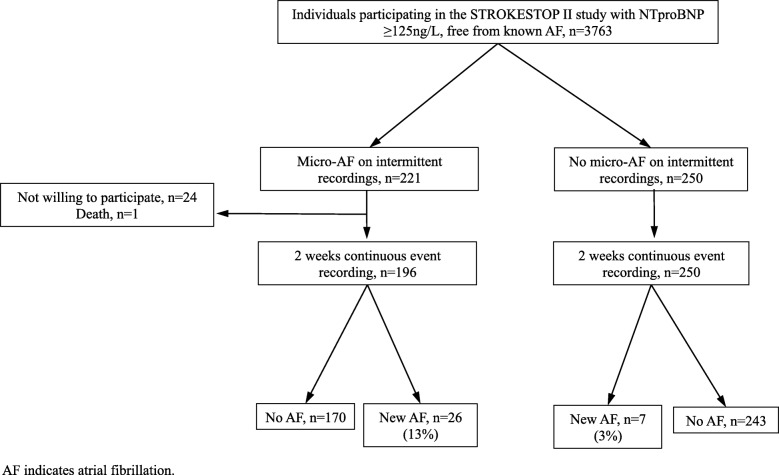
Study flow chart

There were 221 of 3763 (6%) participants with micro-AF in STROKESTOP II. Of those 221 participants, *n* = 196 (89%) participated in this study, Fig. 2.

Participants in the micro-AF group were taller, younger, had lower CHA_2_DS_2_-VASc scores and less frequently had diabetes mellitus compared to the control group, Table 1. There was no difference in NT-proBNP levels. Patients with micro-AF were screened with continuous ECG later than the control group, after 3.3 (IQR 1.8–4.8) months rather than in parallel at zero months (*p* < 0.001).

**Table 1:** Baseline characteristics at study entry

Variable	Micro-AF (*n* = 196)	Control (*n* = 250)	P
Height (cm) median (IQR)	170.0 (165.0–179.0)	169.0 (163.0–177.0)	0.035
Weight (kg) median (IQR)	73.8 (65.0–82.0)	72.0 (63.0–83.0)	0.480
BMI, median (IQR)	24.6 (22.6–27.1)	25.2 (23.0–27.0)	0.436
Systolic BP (mmHg) median (IQR)	138 (129–153)	137 (128–148)	0.107
Diastolic BP (mmHg) median (IQR)	83 (76–90)	82 (74–87)	0.118
Palpitations before study entry, *n* (%)	64 (33)	87 (35)	0.686
NT-proBNP (ng/L) median (IQR)	255 (165–403)	252 (182–363)	0.899
CHA_2_DS_2_-VASc, *n* median (IQR)	3 (3–4)	3 (3–4)	0.019
CHA_2_DS_2_-VASc, *n* mean (95% CI)	3.22 (3.09–3.35)	3.47 (3.34–3.60)	0.008
Congestive heart failure, *n* (%)	4 (2)	4 (2)	0.736
Hypertension, *n* (%)	99 (51)	130 (52)	0.775
Age (years) median (IQR)	76.1 (75.8–76.5)	76.5 (76.2–77.0)	< 0.001
Diabetes Mellitus, *n* (%)	8 (4)	33 (13)	0.001
Stroke/TIA, *n* (%)	11 (6)	22 (9)	0.274
Vascular disease, *n* (%)	9 (5)	21 (8)	0.129
Women, *n* (%)	107 (55)	140 (56)	0.774
Duration between two ECG methods (days) median (IQR)	99 (55–143)	0 (0–0)	< 0.001
Number of intermittent recordings, *n* median (IQR)	53 (46–56)	53 (46–57)	0.969
SVEBs per 30 s intermittent ECG, *n* median (IQR)	0.50 (0.12–1.46)	0.09 (0.02–0.043)	< 0.001

*AF* atrial fibrillation, *BP* blood pressure, *CHA*_*2*_*DS*_*2*_*-VASc* risk score for ischemic stroke, *CI* confidence interval, *IQR* interquartile range, *Micro-AF* short episodes of irregular supraventricular tachycardia, always refers to when it is seen during intermittent ECG recordings, *SVEBs* supraventricular ectopic beats, *TIA* transient ischemic attack

The micro-AF group and the control group underwent similar numbers of ECG recordings, Table 1. The micro-AF group had longer analysed signalling time for the continuous event recorder, 12.6 days (IQR 11.4–13.4) compared to 12.3 days (IQR 10.4–13.3) in the control group, *p* = 0.03.

In the micro-AF group, the median number of micro-AF episodes recorded with intermittent ECG was 1 (IQR 1–1). Most participants had one single micro-AF episode; hence, the number of micro-AF episodes per 30 s ECG were low at 0.02 (IQR 0.02–0.03). Micro-AF episodes were generally short, with an average number of 6 beats (IQR 5–8).

In the micro-AF group, 13% (26/196) had AF detected by extended screening compared with 3% (7/250) in the control group, *p* < 0.001. For participants diagnosed with AF, the median AF burden reported by the software was 1% (IQR 0–4). Of participants diagnosed with AF, 15% (*n* = 5/33) reported typical AF symptoms during their two-weeks registration, although none of them reported symptoms at the exact time of the AF event. All individuals with new AF detected were initiated on OAC therapy. Continuous event recording also detected several other arrhythmias that prompted further investigation. Suspected ventricular tachycardia was detected in 6.7% (*n* = 29/446), 2nd degree atrioventricular block type 2 was found in 2.5% (*n* = 11/446) and 11.4% (*n* = 51/446) of participants had other pauses lasting > 2 s daytime and > 3 s night time.

Individuals diagnosed with AF were taller compared to individuals free from AF and had longer duration of analysed signal time during continuous event recording, Table 2. They also had a higher burden of micro-AF on their intermittent recordings with more frequent micro-AF episodes, micro-AF episodes of longer duration and more SVEBs per intermittent ECG recording, *p* < 0.001 for all comparisons. NT-proBNP was not associated with development of AF.

**Table 2:** Baseline characteristics in participants with new atrial fibrillation diagnosed by continuous ECG monitoring and no atrial fibrillation

	Atrial fibrillation		
Variable	New (*n* = 33)	Never (*n* = 413)	*P*-value
Height (cm) median (IQR)	174 (166–180)	170 (163–178)	0.040
Weight (kg) median (IQR)	74 (70–86)	73 (63.5–82.2)	0.182
BMI, median (IQR)	24.9 (22.6–27.1)	24.9 (22.8–27.0)	0.863
Systolic BP (mmHg) median (IQR)	132 (124–151)	138 (128–150)	0.364
Diastolic BP (mmHg) median (IQR)	82 (79–87)	82 (74–88)	0.601
NT-proBNP (ng/L) median (IQR)	257 (194–432)	253 (175–382)	0.341
CHA_2_DS_2_-VASc, *n* median (IQR)	3 (3–4)	3 (3–4)	0.734
CHA_2_DS_2_-VASc, *n* mean (95% CI)	3.33 (2.93–3.74)	3.36 (3.27–3.46)	0.871
Congestive heart failure, *n* (%)	2 (6)	6 (1)	0.113
Hypertension, *n* (%)	18 (55)	211 (51)	0.722
Age (years) median (IQR)	76.1 (75.8–76.6)	76.4 (76.0–76.8)	0.100
Diabetes Mellitus, *n* (%)	3 (9)	38 (9)	1
Stroke/TIA, *n* (%)	2 (6)	31 (8)	1
Vascular disease, *n* (%)	2 (6)	28 (7)	1
Women, *n* (%)	15 (45)	232 (56)	0.276
Palpitations before study entry, *n* (%)	16 (48)	135 (33)	0.086
SVEBs per 30 s intermittent ECG, *n* median (IQR)	0.89 (0.47–1.86)	0.15 (0.04–0.72)	< 0.001
Number of intermittent recordings, *n* median (IQR)	53 (50–58)	53 (46–56)	0.091
Micro-AF^a^, *n* (%)	26 (79)	170 (41)	< 0.001
Micro-AF episodes, *n* median (IQR)	1 (1–2)	0 (0–1)	< 0.001
Micro-AF episodes per 30 s intermittent recording, *n* median (IQR)	0.02 (0.01–0.03)	0 (0–0.02)	< 0.001
Longest Micro-AF episode (n of complexes) median (IQR)	6 (5–8)	0 (0–6)	< 0.001
Analysed signal time for continuous event recording (days) median (IQR)	13.2 (12.4–13.5)	12.4 (10.8–13.4)	0.013

*AF* atrial fibrillation, *BP* blood pressure, *CHA*_*2*_*DS*_*2*_*-VASc* risk score for ischemic stroke, *CI* confidence interval, *IQR* interquartile range, *Micro-AF* short episodes of irregular supraventricular tachycardia, always refers to when it is seen during intermittent ECG recordings, *SVEBs* supraventricular ectopic beats, *TIA* transient ischemic attack

^a^ For all variables including micro-AF, intermittent recordings are assessed

**Fig. 3:**
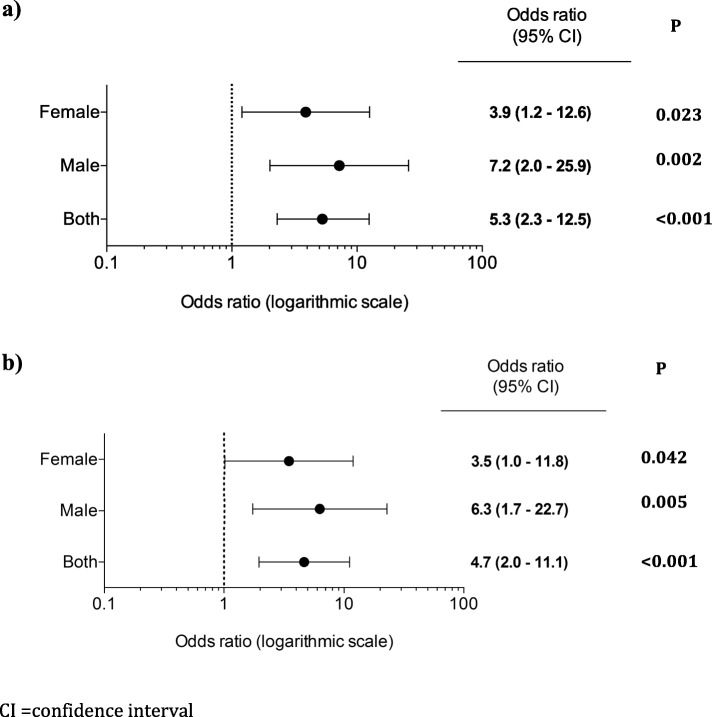
Micro-atrial fibrillation as a risk factor for AF depending on gender, presented in odds ratios. **a** Unadjusted analysis. **b** Adjusted for analysed signal time and height

Presence of micro-AF remained associated with an increased risk for AF in the multivariable analysis (OR 5.1 (95% CI 2.1–12.8), Table 3. In a stratified analysis, micro-AF was shown to be a stronger predictor for AF in males than in females, Fig. 3.

**Table 3:** Multivariable analysis for the development of atrial fibrillation

			Model 1		Model 2		Model 3	
Variable	Unadjusted OR (CI 95%)	P	Adjusted OR (CI 95%)	P	Adjusted OR (CI 95%)	P	Adjusted OR (CI 95%)	P
**Micro-AF**	5.3 (2.3–12.5)	< 0.001	4.7 (2.0–11.1)	< 0.001	5.1 (2.1–12.8)	< 0.001	4.6 (1.8–11.5)	< 0.001

Model 1: Adjusted for height and analysed signal time

Model 2: Adjusted for age, hypertension, heart failure and stroke

Model 3: Adjusted for height, analysed signal time, age, hypertension, heart failure and stroke

*AF* atrial fibrillation, *CI* confidence interval, *Micro-AF* short episodes of irregular supraventricular tachycardia, always refers to when it is seen during intermittent ECG recordings; *OR* odds ratio, *SVEBs* supraventricular ectopic beats

## Discussion

The main finding in this study is that 13% of individuals with micro-AF seen during intermittent recordings had AF detected by extended screening using continuous event recording. AF prevalence was found to be more than four times higher in the micro-AF group (13%) compared to the control group (3%).

In a study by Binici et al., healthy individuals aged 55–75 years old underwent 48-h ECG monitoring, analysed for SVTs (≥20 beats) and ≥ 30 SVEBs per hour. After 6.3 years register follow-up, they were shown to have a 3-fold risk of AF and a 60% increased risk of stroke and death compared to controls. A linear association was seen between AF development and both frequency of SVEBs and length of SVTs [8]. Frequent isolated SVEBs and SVTs during 24-h ECG monitoring also predisposed for AF and stroke [9, 10].

In a Swedish cohort study, individuals free from AF with a mean age of 64.5 years underwent 24-h ECG monitoring and were followed prospectively for > 13 years. SVTs with different characteristics were compared; irregular SVTs without P waves showed the strongest association with clinical AF, with a cumulative incidence of 47.4% [15]. For irregular SVTs without P waves, a similar classification as our micro-AF definition was used.

The mean duration of irregular SVTs without P waves in the Swedish cohort study was 13 beats, compared to six in our study. This may be due to shorter monitoring time in our study. Interestingly, most episodes are very short. Indeed there were few episodes in the range approximating 30 s. It seems that episodes are either shorter than 10 s, or long enough to fulfill the AF criterion. This could possibly be explained by the fact that once an episode becomes long enough it will continue to trigger arrhythmia.

It is not known if the stroke risk in individuals with excessive supraventricular activity is increased independently of AF or if the increase is due to undetected AF. Most prior studies have been done with register follow-up, where asymptomatic patients with AF are likely to remain unaccounted for as they are less likely to be diagnosed. More studies using active AF screening during follow-up is needed to determine the stroke risk in individuals with SVTs.

The risk of stroke with paroxysmal AF is high even during sinus rhythm [21], making mechanisms other than impaired hemodynamic of the left atrium during AF contributing to thrombus formation likely. Extensive atrial fibrosis has a role in the prothrombotic stage associated with AF but also isolates myocytes, creating an environment where re-entry arrhythmia, like atrial fibrillation, can exist [22–24]. This suggests that AF, SVTs and SVEBs could be signs of atrial cardiomyopathy and might thereby be independently associated with an increase in stroke risk. Interestingly, in our study we could not see a significant increase in NT-proBNP level neither in individuals with micro-AF nor AF. The lack of significant increase could possibly be due to low burden of both micro-AF and AF in our study participants or be explained by that the NT-proBNP cut-off level ≥ 125 ng/L may already be too high to detect smaller differences.

In our previously published pilot-study we retrospectively examined a subset from a different cohort, STROKESTOP I. We invited participants with micro-AF (*n* = 47) to a repeat AF screening procedure 2.3 years after the first screening. The repeat screening was done with continuous-event recorder over a two-week period. New AF was found in 50% of individuals who had shown short irregular SVEB runs of ≥4 beats without P waves on the first screening, compared to only 10% in the control group [25]. In this study no initial prolonged ECG monitoring was performed, leaving uncertainty as to whether micro-AF episodes were just a sign of previously underdetected AF or a risk marker for developing AF. The AF prevalence found in participants with micro-AF was four times higher after 2.3 years in our pilot-study compared to after 3.3 months in our current study, indicating that micro-AF is a risk marker for AF but also a sign for already existing undetected AF.

AF is a progressive disease which begins with short infrequent episodes and evolves to more permanent forms over time [26]. Most earlier studies have been registry studies with a long follow-up time to clinically detect AF [8, 9]. In our study after a median of 3.3 months, 13% of participants with micro-AF were found to have AF.

Early diagnosis of AF is important in populations with high risk of stroke in order to initiate preventive treatment before stroke and other complications of AF occur. Our study results indicate that high-risk individuals with micro-AF may benefit from extended and early AF screening. This confirms earlier findings that repeat AF screening after 2 years in this group may be beneficial [25]. Our study was not powered to detect associations between micro-AF and thromboembolic risk; further studies are needed to study the association. It is not known if high-risk individuals with micro-AF would benefit from OAC. However, those individuals could benefit from risk free interventions, primary prevention, optimising lifestyle factors and treating co-morbidities as an effort to reverse atrial myopathy. Primary prevention in AF is discussed in more detail in a summary of the Heart Rhythm Society Research Forum [27].

One limitation in our study is that the micro-AF group underwent continuous event recording in a median of 3.3 months later than the control group. It is possible that this introduced detection bias, and more AF cases might have been found in the control group using the same follow-up time. This could lead to overestimation of our findings.

Both the Zenicor device and the Rtest4 are 1-lead ECG devices which sometimes make detection of atrial activity challenging. This could possibly lead to misdiagnosis of both AF and atrial flutter and introduce a misclassification bias by underestimation of true cases. This potential misdiagnosis, however, is likely to affect the micro-AF group more as the prevalence of atrial fibrillation is higher in this group and would have the potential to reduce the significance of our findings.

According to our experience, the R-test 4 has a propensity to over-diagnose AF. Only a limited number of episodes with AF suspicious activity are stored for manual inspection. Due to low specificity, all episodes marked as AF by the device need to be manually verified. It is therefore possible that some AF episodes may have been missed due to device memory limitations. The R-test 4 system does not store the full disclosure ECG of all episodes marked as AF, making it difficult to estimate the true AF burden. The limited duration of the screening period also introduces a risk for under-diagnosis of AF.

All participants were initially part of STROKESTOP II. They were a selected group, all born in 1940 or 1941. The majority were Caucasians. It is possible that the participants are healthier than the general population, as individuals participating in screening studies tend to be more health conscious than non-participants [28]. Also, only participants from STROKESTOP II with elevated NT-proBNP levels were eligible for the study. This could lead to over-estimation of the micro-AF prevalence and may also affect the external validity of the study. Micro-AF may also indicate a higher risk for AF in individuals with increased NT-proBNP levels, than in individuals with normal levels.

## Conclusions

The presence of very short-lasting episodes of AF-like activity, termed micro-AF, indicates high risk for undetected AF. Continuous ECG screening should be recommended if a finding of AF would lead to initiation of stroke preventive OAC treatment.

## Supplementary information

**Additional file 1:Table S1.** Settings for number of ECG findings stored by R-test 4.

## Data Availability

The datasets used and/or analysed during the current study are available from the corresponding author on reasonable request. The dataset is planned to be used in future studies.

## Abbreviations

AF: Atrial fibrillation
BP: Blood pressure
CHA_2_DS_2_-VASc: Risk score for ischaemic stroke
CI: Confidence interval
IQR: Interquartile range
Micro-AF: Short episodes of irregular supraventricular tachycardia
OAC: Oral anticoagulation
OR: Odds ratio
SVTs: Supraventricular tachycardias
SVEBs: Supraventricular ectopic beats
TIA: Transient ischaemic attack
