# Supplementary material for: Brief episodes of rapid irregular atrial activity (micro-AF) are a risk marker for atrial fibrillation: a prospective cohort study
Source: BMC Cardiovasc Disord. 2020 Apr 10;20:167. doi: 10.1186/s12872-020-01453-w (PMC7149874; doi:10.1186/s12872-020-01453-w)
Supplement: Supplementary file 1 — Additional file 1:Table S1. Settings for number of ECG findings stored by R-test 4. [file 12872_2020_1453_MOESM1_ESM.docx]

**Supplementary table 1.** Settings for number of ECG findings stored by R-test 4

| Arrhythmia | Number of episodes |
| --- | --- |
| Ventricular tachycardia  Ventricular ectopic beats  Paroxysmal supraventricular tachycardia  Supraventricular ectoptic beats  Absolute paus  Relative paus  Tachycardia  Bradycardia  ST-changes  Atrial fibrillation  Marker (by the patient) | 4  4  10  5  10  4  8  3  0  42  8 |
